# Outpatient Service Use in Korean Older Adult Women with Degenerative Arthritis Based on Andersen’s Model

**DOI:** 10.3390/geriatrics8010009

**Published:** 2023-01-06

**Authors:** Soyoung Jang, Eunyoung E. Suh

**Affiliations:** 1 Center for Human-Caring Nurse Leaders for the Future by Brain Korea 21 (BK 21) Four Project, College of Nursing, Seoul National University, Seoul 03080, Republic of Korea; 2 Research Institute of Nursing Science, College of Nursing, Seoul National University, Seoul 03080, Republic of Korea

**Keywords:** outpatient health services, older adult, women, degenerative arthritis

## Abstract

To ensure that older adults (aged 65 years or older) can experience a healthy life, they should use medical services that are appropriate, both quantitatively and qualitatively. This study aimed to identify the factors affecting outpatient service use by older adult women with degenerative arthritis using Andersen’s model. A survey was conducted among 232 older adult women with degenerative arthritis in two university hospitals in Seoul. The Korean Activities of Daily Living, Korean Instrumental Activities of Daily Living, and the Geriatric Depression Scale Short Form were used. Data were analyzed using descriptive statistics, χ^2^-test, *t*-test, and multiple logistic regression analysis. Among the participants, 69.8% used outpatient services and 30.2% did not. In the univariate analyses, age, marital status, residency, household income, chronic diseases, subjective health status, and disability were significant. Age (odds ratio [OR] = 5.53, *p* < 0.001), annual household income (OR = 5.64, *p* < 0.001), chronic diseases (OR = 11.06, *p* < 0.001), and disability (OR = 3.56, *p* = 0.016) significantly affected outpatient service use. The results suggest that health promotion interventions for Korean older adult women should focus on predicting outpatient service use according to the patient’s characteristics.

## 1. Introduction

With the extension of life expectancy in Korea, the proportion of older adults (aged 65 years or older) has increased by five times since 1970, having reached 14.9% in 2019, and is expected to reach 24.3% by 2030 and 46.5% by 2067 [1]. Moreover, by 2025, Korea is expected to become a super-aged society: that is, adults aged 65 or older will exceed 20% of the population [2]. Thus, the prospective demand for medical services will steadily increase because of the physical and mental changes that older adults experience through the aging process [3,4]. Indeed, older adults often incur health problems that negatively affect their quality of life, such as chronic diseases and their related symptoms and limitations in physical functions [5,6]. Moreover, globally, the number of older adults living longer and with various chronic diseases is increasing [7,8]. Therefore, to ensure that people can experience healthy old age, older adults should use medical services that are appropriate, both quantitatively and qualitatively, to their unique characteristics.

Degenerative arthritis is one of the most common chronic diseases in older people; it causes gradual damage or degenerative changes in the cartilage that protect the joints, thereby damaging the bones and ligaments of a joint and leading to inflammation and pain [9]. In South Korea, the prevalence of degenerative arthritis has increased in those aged 50 years or older, appearing in 25.3% of those in their 60s and 41.5% of those in their 70s; the prevalence of this disease in women is about four times higher than that in men [10]. This disease can easily be deemed a serious health problem for older people as it limits movements in daily living, degrades the quality of life owing to physical changes, and generates high medical expenses [11,12]. 

Outpatient service use by older adults in Korea has increased 3.8 times from 1990 to 2010 (i.e., from 6.8% to 25.7%), indicating that this population has grown to account for about a quarter of all outpatient service use nationwide [13]. Given the rapid aging phenomenon, the steady increase in medical service use, and the remarks of prior research [14,15], it seems that identifying the influencing factors of outpatient service use in Korean older people may increase our general understanding of medical service use. It also has the potential of providing valuable data for political and managerial policymakers that help to reduce the inequality in access to healthcare nationwide. 

The literature shows that Andersen’s behavioral model is a widely used framework for examining the determinants of medical service use in older adults [16]; it categorizes the influencing factors of medical service use into predisposing, enabling, and need factors [17]. Specifically, this model is useful for identifying the health, sociodemographic, and economic characteristics associated with older adults’ health status [17]. Despite the utility of this model for research, there seems to be a lack of studies applying it to predicting medical service use in Korea, and this lack becomes most evident when contrasted with the number of international studies on the topic [18,19,20,21]. 

One of the few domestic studies that used Andersen’s model on an older adult sample showed that, in the short term, the need factors (e.g., chronic diseases and disabilities) have particularly significant effects, with chronic disease being a causative factor increasing outpatient service use [14]. Lee [22] conducted a longitudinal study using a Korean sample and showed that the predisposing factors (e.g., not living with children, the female sex, and face-to-face contact with others) and enabling factors (e.g., household income and residency in urban areas) were associated with high medical service use. Subsequently, a study published in 2018 showed that outpatient service use rates increased with a negative subjective perception of one’s health status and with the presence of multiple chronic diseases [3]. Another study on outpatient service use and health behavior in Korea showed that living in urban areas, being overweight, having a higher income, and drinking alcohol led to higher medical service use rates [23]. 

Particularly, some domestic studies have shown that sex affects outpatient service use in older people [15,22]. In 2015, in Korea, older adult women aged 65 or older accounted for 71.7% of the older adult population, showing that there is a higher proportion of older adult women than men, that they are aging faster than men, and that their life expectancy is 4.4 years higher than that of men [24]. Concomitantly, Korean older adult women were reported to have lower values for physical, mental, and economical factors when compared to men [25]. Furthermore, older adult women reportedly have a higher prevalence of chronic diseases and a higher risk of cognitive impairment than older adult men [26]; this last cited study also showed that depressive symptoms and morbidity rates were about two times higher, and mobility limitation was three times higher, in older adult women than in their male counterparts. Moreover, older adult women use outpatient services more than older adult men because they are more vulnerable to low economic income, poor physical and mental health, react more sensitively to stress, and perceive their health conditions more negatively [26].

In summary, thus far, few studies have analyzed medical service use in Korean older adult women using Andersen’s model. Nonetheless, against the increasing importance of the health of this population owing to the aging population phenomenon, we have been observing a steady surge in the number of studies on older adult health. Despite such augmentation, few studies have been devoted to examining the factors associated with outpatient service use within the context of specific chronic diseases [16].

**Figure 1:**
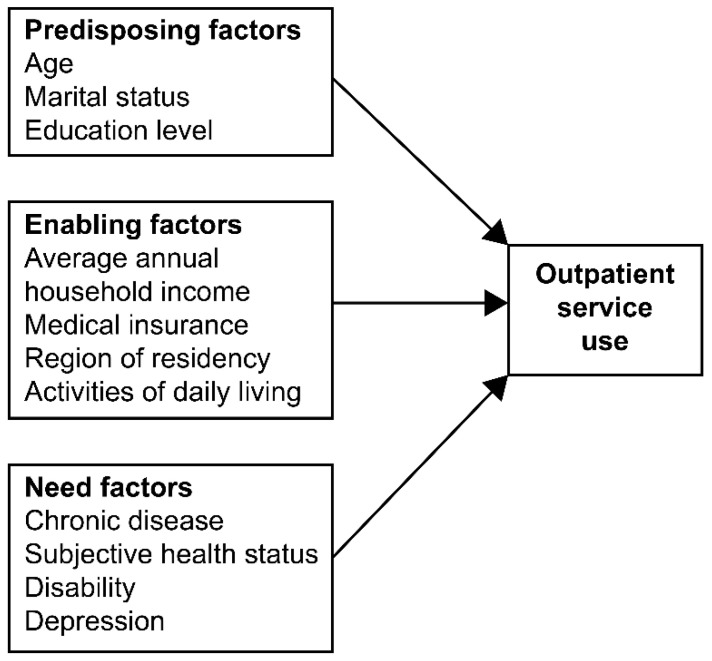
The modified model based on Andersen’s model.

Therefore, this study aimed to analyze the factors affecting outpatient service use by Korean older adult women with degenerative arthritis using Andersen’s model. The final aim of this study was to help enable Korean older adult women to lead healthier lives and experience greater societal equality regarding access to healthcare. In addition, data on outpatient service use patterns may prove useful for stakeholders in the development of interventions for improving the health of older adult women with degenerative arthritis, as well as assist those interested in improving resource allocation in medical settings in Korea. In this study, a conceptual framework for outpatient service use based on Andersen’s model was proposed, which is shown in Figure 1.

## 2. Methods

### 2.1. Study Design

This was a descriptive, cross-sectional study.

### 2.2. Samples

The inclusion criteria of this study were being of the female sex, aged 65 years or older, having degenerative arthritis, and having visited the outpatient department of two university hospitals in Seoul, Korea. Those under 65 years of age were excluded. To determine the number of participants required for logistic regression analysis, the G*power version 3.1 program (Heinrich Heine Universität Düsselforf, Schwaderer, Germany) was used with the significance level set to 0.05, an effect size for odds ratio (OR) of 1.72, and the power set at 0.8. The results showed an ideal sample size of 177. The final valid sample of 232 participants was deemed sufficient to ensure statistical power.

### 2.3. Data Collection

The researchers submitted a research plan to the institutional review boards of the two university hospitals with information on the study purpose and procedures. The study was reviewed and approved by the Biomedical Research Institute of the Seoul National University Hospital and the Institutional Review Board of the Chung-Ang University Hospital (approval numbers: H-1703-019-836, 1740-006-278, respectively).

Data were collected from 3 April to 27 May 2017. Prior to participation, all potential study participants received explanations of the study’s purposes and procedures, the fact that all personal information collected would be kept confidential, and that participation was voluntary. All participants provided verbal informed consent. This research used a self-reported questionnaire, but whenever participants presented any difficulties in filling out the questionnaire, the researcher read the questions to them aloud, to which study participants answered. In total, 250 questionnaires were distributed, 248 (99%) were returned, and 232 (93%) were finally used for data analysis. Data from 16 questionnaires were deleted because they had items left blank, hindering the ability to use them in the statistical analysis process.

### 2.4. Measurements

The questionnaire used in this study comprised 43 questions: 9 on sociodemographic characteristics, 7 on activities of daily living (ADL), 10 on instrumental activities of daily living (IADL), 15 on depression, and 2 on outpatient service use.

#### 2.4.1. Activities of Daily Living

The 7-item K-ADL, a tool modified by Won et al. [27] for suitability with the Korean culture based on Katz et al. [28], was used to assess ADL. It includes one item each for dressing, bathing, eating, toileting, transfer, continence, and washing the face and hands, and it is answered on a 3-point scale (1, perfect self-reliance; 2, partial dependence; 3, complete dependence). Total scores range from 7 to 21 points, where the higher the score, the higher the dependence. In Won et al.’s [27] study, the Cronbach’s α of this scale was 0.94; in this study, it was 0.78.

#### 2.4.2. Instrumental Activities of Daily Living

The 10-item K-IADL, a tool modified by Won et al. [27] for suitability with the Korean culture based on Lawton and Brody [29], was used to assess I-ADL. It includes one item each for grooming, housework, preparing meals, laundry, short trips, using transportation, shopping, managing money, using the telephone, and taking medicine, and it is answered on a 3-point scale (1, perfect self-reliance; 2, partial dependence; 3, complete dependence). Total scores range from 10 to 30 points, where the higher the score, the higher the dependence. In Won et al.’s [27] study, the Cronbach’s α of this scale was 0.94; in this study, it was 0.91.

#### 2.4.3. Depression

To measure the level of depression in older adults, the 15-item Korean version of the Geriatric Depression Scale, which was adapted by Kee [30] based on the Short Form Geriatric Depression Scale developed by Yesavage et al. [31], was used. Items are answered on a dichotomous scale (1, yes; 0, no), with total scores ranging from 0 to 15 points. For this tool, a score within the range of 0–4 points is considered as indicating a healthy mental status, 5–9 points as mild depression, and 10–15 points as severe depression. Moreover, 5 of the 15 questions were negative, so they required reverse coding, with an answer of “no” counting as 1 point. In Kee’s [30] study, the Cronbach’s α of this scale was 0.88; in this study, it was 0.63. 

Of note, in this study, the participants’ responses regarding their mood conditions may not be consistent because of the varying severity of the disease in the sample, which comprised older adult female outpatients with degenerative arthritis. However, the Cronbach’s α of the scale for this study was still deemed as a valid descriptor of the reliability of the scale.

### 2.5. Data Analysis

For data analysis, the SPSS program, version 24.0 (IBM Corp., Armonk, NY, USA), was used. The predisposing, enabling, and need factors were analyzed using frequency, percentage, mean, and standard deviation. The differences in outpatient service use according to the three factors were analyzed using χ^2^ test or *t*-test. To analyze the factors affecting outpatient service use by the three types of factors, multiple logistic regression analysis was used. In this study, the variance inflation factor among independent variables was checked. It confirmed that there were no multicollinearity problems among the study variables.

## 3. Results

### 3.1. Demographic Characteristics

In total, there were 232 participants, and all were older adult women; 162 (69.8%) of them used outpatient services from 1 March 2016 to 28 February 2017. The average frequency of outpatient service use in the past year was eight times.

The results of analyzing the participants’ sociodemographic characteristics according to the predisposing, enabling, and need factors are shown in Table 1. Regarding age, there were 168 (72.4%) younger-older adults (i.e., aged 65–75 years) and 64 (27.6%) older-older adults (i.e., aged 75 years or older); the average age was 72.11 years. For average annual household income, 126 (54.3%) participants earned less than 30 million won, with an average of 33.5 million won. In total, 182 (78.4%) participants had other chronic diseases aside from degenerative arthritis. 

### 3.2. Differences in the Predisposing, Enabling, and Need Factors Related to Outpatient Service Use

The results of analyzing differences in outpatient service use by the participants’ predisposing, enabling, and need factors are shown in Table 2. Among the predisposing factors, regarding age, 73.8% of the younger-older adults and 59.4% of the older-older adults used outpatient services; the difference between them was statistically significant (χ^2^ = 4.58, *p* = 0.038).

Among the enabling factors, regarding the region of residency, 76.7% of those living in Seoul and 60.6% of those in other metropolitan cities/provinces used outpatient services; the difference between them was statistically significant (χ^2^ = 6.97, *p* = 0.009). 

Among the need factors, regarding other chronic diseases aside from degenerative arthritis, 75.8% of those with other chronic diseases and 48.0% of those without other chronic diseases used outpatient services; the difference between them was statistically significant (χ^2^ = 14.41, *p* < 0.001). 

Regarding disability, 75.1% of those without a disability and 35.5% of those with a disability used outpatient services; the difference between these groups was statistically significant (χ^2^ = 20.03, *p* < 0.001). 

Finally, there was no significant difference between groups in terms of depression (t = 0.58, *p* = 0.566), with a mean score of 8.57 (±3.20) for those who used outpatient services and a score of 8.31 (±2.78) for those who did not.

### 3.3. Factors Influencing Outpatient Service Use

The results of the multivariate logistic regression analyses for the factors affecting the participants’ outpatient service use are shown in Table 3. The result regarding −2 log-likelihood was 179.87, and the explanatory power of the data, demonstrated using Nagelkerke R^2^, was 0.36.

In Model 3, being a younger-older adult (OR = 5.53, *p* < 0.001) and having an average annual household income of more than 30 million won (OR = 5.64, *p* < 0.001), other chronic diseases aside from degenerative arthritis (OR = 11.06, *p* < 0.001), and no disability (OR = 3.56, *p* = 0.016) showed a significant effect on outpatient service use. Moreover, those with other chronic diseases aside from degenerative arthritis had an OR of 11.06 times higher than that of participants without other chronic diseases, and participants without disabilities had an OR of 3.56 times higher than that of individuals with a disability.

## 4. Discussion

Using Andersen’s model, this study analyzed the effects of predisposing, enabling, and need factors on outpatient service use by Korean older adult women with degenerative arthritis. The results showed that age (predisposing factor), average annual household income (enabling factor), and other chronic diseases aside from degenerative arthritis and disability (need factors) affected outpatient service use. 

First, the difference between age groups was statistically significant, with 73.8% of the younger-older adults and 59.4% of the older-older adults using outpatient services. This is consistent with prior studies, which showed that outpatient service use rates were higher among younger-older adults [32,33]. Hence, it is expected that the younger the older adult, the better the health functioning, and the higher the outpatient service use rate; this may owe to younger-older adult women with degenerative arthritis experiencing less physical discomfort amid the processes required for using outpatient services (e.g., traveling to the medical institution).

Research has shown that as people age (mean age 76.0 ± 6.9), the fewer preventive services they utilize, and the less they talk about their health problems to medical staff, which may be because of the patient’s acceptance of their health deterioration, deeming it as a natural aging phenomenon and that health status restoration is a non-important issue [34]. Therefore, it seems necessary to encourage older adult women with degenerative arthritis to reduce their medical costs by implementing preventive efforts, which can be operationalized using the delivery of health checkup services to help them recognize the seriousness of the disease.

Second, married participants showed higher outpatient service use rates. This finding is similar to prior evidence, which showed that the higher the support from the family, the higher the use of outpatient services [35,36]. Nevertheless, the finding of the current study differs from prior research that applied Andersen’s model and that was conducted with Korean older adults, which showed that people who do not live with their children are more likely to use outpatient services [3]. These results may be because, although older adult women may desire to receive medical services, those who lack family support may be left unattended because they are hindered in their accessibility to healthcare services. Therefore, it may be necessary to increase accessibility support for outpatient services among Korean older adult women with degenerative arthritis who do not live with their spouses or children.

Third, the region of residency had a statistically significant effect on outpatient service use. Prior research showed that the reason behind the influence of the region of residency on medical service use may lie in its relationship with the medical environment, including the reasons why people access and search for medical institutions, the area of residency, and the number of hospitals in the region [37]. This finding is also consistent with the finding that older adult women with degenerative arthritis who live in urban areas spend more money on healthcare [21]. 

Fourth, the results showed that an average annual household income of 30 million won or more had a statistically significant effect on outpatient service use, as those with such income showed higher use. This finds consistency with the results of a study that applied Andersen’s model [3]. In another study, it was shown that older women with higher average annual household income actively accepted health information and were more willing to directly practice the information they received, while their counterparts with lower average income more restrictively engaged in changing their health behaviors owing to daily life-related limitations and stress [38]. 

Fifth, a major variable affecting outpatient service use in the current study was having other chronic diseases aside from degenerative arthritis, as those with multiple chronic diseases showed higher outpatient service use. This finding concurs with the literature, which shows that older adult women with various chronic diseases have higher outpatient service use rates [3,21,39,40]. The fact that older adults have many chronic diseases may lead them to desire to receive various, and often complex, medical services, making a higher outpatient service use rate a natural consequence of such desire/services [33,41]. Therefore, older adult women with degenerative arthritis may require the delivery of continuous health checkup services, personalized treatment, and disease management. For this, it may be necessary to provide them with preventive medical services and long-term disease management, which can be operationalized using the promotion of a continuous connection between patients and outpatient services at medical institutions.

Sixth, older adult women with degenerative arthritis who did not have a disability showed higher outpatient service use, and this was a major affecting variable. It is assumed that the outpatient service use rate was low among those with disabilities because they may feel more uncomfortable performing their ADL. In a study using Andersen’s model, conducted with pre-older adults with disability, outpatient service use was found to be very high in patients with chronic diseases [42]. Although a direct comparison between the current study and this cited study is difficult owing to sample differences, this still indicates that the need factor has a significant effect on outpatient service use. 

Lastly, our study showed that older women with degenerative arthritis who used outpatient services had slightly higher depression levels than those who did not. A meta-analysis reported that some chronic diseases including arthritis were related to risk factors for depression in older adults [43]. This is believed to be because the proportion of older adult women with chronic disease who used outpatient services was higher than those who did not. Depression is associated with chronic diseases and is one of the major factors in healthcare utilization and costs [44]. Therefore, more attention needs to be diverted toward preventing and managing chronic diseases in older adults. 

In Model 3 of the logistic regression analysis of the current study, the influencing factors of outpatient service use were age, average annual household income, having other chronic diseases aside from degenerative arthritis, and disability. Moreover, the need factors had more influence than the predisposing factors and enabling factors. These results were consistent with those from two other studies, which showed chronic disease as an important factor influencing medical service use in older adults [3,21].

### 4.1. Limitations

First, as this cross-sectional study surveyed only older adult women who frequented two university hospitals in Seoul, generalizations should be made with caution. Second, because this study included a self-reported questionnaire, attention should be paid to potential objectification. Future research should use qualitative (e.g., interviews and participant observation) and quantitative research methods concomitantly, as this will enable a more comprehensive understanding of the studied phenomena and enrich the current findings. Finally, this cross-sectional survey study collected data over a specific, short-term period; future studies should longitudinally analyze the factors affecting outpatient service use.

### 4.2. Theoretical Implications 

Since Andersen’s model is an integrated model that considers internal and external factors at the individual level, it has been frequently applied in research on medical service use, as mentioned beforehand. In this study, its use was meaningful in that it allowed for the identification of the factors affecting outpatient service use in Korean older adult women with chronic diseases, a setting in which the application of this model for the analyses of the aforementioned variables has rarely occurred. These data may be useful for stakeholders who research the variables related to healthy living in Korean older adult women and may eventually translate into actual healthier lives for this population. 

### 4.3. Practical Implications

The findings and discussions in this study suggest the need for improvements in interventional strategies regarding access to outpatient service use for those who are underprivileged. First, the national government should come up with measures to improve the medical service support system, allowing older-older adults to receive continuous health checkups and individualized disease management services. Second, among older adults with disabilities, it may be necessary to expand policy support for hospital accompaniment services in order to ensure that this population can access outpatient care. 

Third, it seems essential to expand the policies on visiting care for chronic disease prevention and/or to develop and apply nursing intervention programs related to such prevention measures. Finally, the data from this paper may prove meaningful to help older adult women in Korea experience greater social equality in access to healthcare.

## 5. Conclusions

In this study conducted with 232 Korean older adult women with degenerative arthritis, Andersen’s model was used to analyze the factors affecting outpatient service use. In particular, the factors with the largest influence were the need factors. Hence, and confirming prior literature [16], the need factors in Andersen’s model were major factors influencing outpatient service use. This scientific assumption may also be applicable to Korean older adult women with degenerative arthritis. 

The results of this study suggest that health promotion interventions for Korean older adult women should be devoted to predicting outpatient service use according to the patient’s characteristics. For the future, we recommend studies that can reproduce our analyses while using larger samples and longitudinally analyzing the study variables.

## Figures and Tables

**Table 1:** Participants’ sociodemographic characteristics.

Factors	Variables	Categories	*n* (%)	M ± SD
Outpatient service use		Yes	162 (69.8)	
		No	70 (30.2)	
Predisposing factors	Age (y)	<75	168 (72.4)	72.11 ± 5.68
		≥75	64 (27.6)	
	Marital status	Married	172 (74.1)	
		Others	60 (25.9)	
	Education level	Less than secondary school	105 (45.3)	
		High school or higher	127 (54.7)	
Enabling factors	Region of residency	Seoul	133 (57.3)	
		Others	99 (42.7)	
	Medical insurance	Health insurance	210 (90.5)	
		Medical benefit	22 (9.5)	
	Average annual household income (in won)	<30 million	126 (54.3)	3350.56 ± 4408.09
		≥30 million	106 (45.7)	
	ADL			7.22 ± 0.86
	IADL			11.11 ± 2.77
Need factors	Chronic disease	Yes	182 (78.4)	
		No	50 (21.6)	
	Subjective health status	Healthy	134 (57.8)	
		Not healthy	98 (42.2)	
	Disability	Yes	31 (13.4)	
		No	201 (86.6)	
	Depression			8.49 ± 3.08

*N =* 232. ADL = activities of daily living; IADL = instrumental activities of daily living.

**Table 2:** Differences in outpatient service use by predisposing, enabling, and need factors.

Factors	Variables	Categories	Outpatient Service Use		χ^2^/t	*p*
			Yes (*n* = 162) *n*(%)/M ± SD	No (*n* = 70) *n*(%)/M ± SD		
Predisposing factors	Age (y)	<75	124 (73.8)	44 (26.2)	4.58	0.038
		≥75	38 (59.4)	26 (40.6)		
	Marital status	Married	127 (73.8)	45 (26.2)	5.08	0.033
		Others	35 (58.3)	25 (41.7)		
	Education level	Less than secondary school	74 (70.5)	31 (29.5)	0.04	0.886
		High school or higher	88 (69.3)	39 (30.7)		
Enabling factors	Region of residency	Seoul	102 (76.7)	31 (23.3)	6.97	0.009
		Others	60 (60.6)	39 (39.4)		
	Medical insurance	Health insurance	148 (70.5)	62 (29.5)	0.44	0.626
		Medical benefit	14 (63.6)	8 (36.4)		
	Average annual household income (in won)	<30 million	78 (61.9)	48 (38.1)	8.22	0.004
		≥30 million	84 (79.2)	22 (20.8)		
	ADL		7.15 ± 0.61	7.39 ± 1.27	−1.46	0.148
	IADL		10.99 ± 2.70	11.39 ± 2.93	−1.00	0.316
Need factors	Chronic disease	Yes	138 (75.8)	44 (24.2)	14.41	<0.001
		No	24 (48.0)	26 (52.0)		
	Subjective health status	Healthy	101 (75.4)	33 (24.6)	4.63	0.042
		Not healthy	61 (62.2)	37 (37.8)		
	Disability	Yes	11 (35.5)	20 (64.5)	20.03	<0.001
		No	151 (75.1)	50 (24.9)		
	Depression		8.57 ± 3.20	8.31 ± 2.78	0.58	0.566

*N =* 232. ADL = activities of daily living; IADL = instrumental activities of daily living.

**Table 3:** Multiple logistic regression analysis results for the factors affecting outpatient service use.

Categories	Model 1					Model 2					Model 3				
	B	SE	*p*	OR	95% CI	B	SE	*p*	OR	95% CI	B	SE	*p*	OR	95% CI
**Age**															
≥75				1					1					1	
<75	1.00	0.34	0.003	2.72	1.40–5.29	1.09	0.36	0.003	2.96	1.46–6.00	1.71	0.45	<0.001	5.53	2.31–13.24
**Marital status**															
Others				1					1					1	
Married	0.62	0.35	0.078	1.86	0.93–3.70	0.37	0.37	0.320	1.45	0.70–3.00	0.37	0.41	0.371	1.44	0.65–3.24
**Region of residency**															
Others									1					1	
Seoul						0.44	0.35	1.611	0.20	0.79–3.06	0.47	0.38	0.222	1.59	0.75–3.37
**Average annual household income**															
<30 million									1					1	
≥30 million						1.46	0.40	<0.001	4.30	1.97–9.35	1.73	0.44	<0.001	5.64	2.39–13.30
**Chronic disease**															
No														1	
Yes											2.40	0.49	<0.001	11.06	4.24–28.88
**Subjective health status**															
Not healthy														1	
Healthy											0.43	0.39	0.277	1.53	0.71–3.32
**Disability**															
Yes														1	
No											1.27	0.53	0.016	3.56	1.27–9.99
−2Log- Likelihood	228.70					211.35					179.87				
Cox and Snell R^2^	0.06					0.12					0.23				
Nagelkerke R^2^	0.09					0.19					0.36				

OR = odds ratio; CI = confidence interval.

## Data Availability

The datasets used and/or analyzed during the current study are available from the corresponding author on reasonable request.
